# Psychometric properties of the simplified Chinese version of the observer OPTION^5^ scale

**DOI:** 10.1186/s12875-020-01335-2

**Published:** 2020-12-06

**Authors:** Zhaojuan Chen, Xuefei Bai, Guanghui Jin, Xin Tao, Guowei Huang, Yali Zhao

**Affiliations:** 1 School of General Practice and Continuing Education, Capital Medical University, No. 10, Xitoutiao, You An Men Wai, Fengtai District, Beijing, 100069 China; 2 Hai Dian District Shuangyushu Community Health Service Center, Beijing, China; 3 School of Medical Humanities, Capital Medical University, Beijing, China

**Keywords:** Shared decision-making, Patient involvement, Communication, OPTION^5^, Measurement, Psychometric properties

## Abstract

**Background:**

OPTION^5^ is a scale used to evaluate shared decision making (SDM) in health care from an observer’s perspective; however, to date, there is no simplified Chinese version of this scale.

**Objectives:**

This study aims to produce a simplified Chinese version of the OPTION^5^ scale and to test its psychometric properties.

**Methods:**

One rater observed and audio-recorded consultations between general practitioners (GPs) and chronically ill patients in a Beijing community health service center (CHSC) from May to June 2019. Meanwhile, demographic data of the patients and GPs were collected via information forms. Two raters assessed inter- and intra-rater reliability by calculating the intraclass correlation coefficient (ICC) and weighted Cohen’s Kappa values. Internal consistency was assessed using Cronbach’s α value. Concurrent was calculated by Spearman’s rank correlation coefficient.

**Results:**

A total of 209 consultations were recorded and evaluated. As concerns inter-rater reliability, the ICC of the OPTION^5^ was 0.859 on the total score level, with Cohen’s weighted k ranging from 0.376 (item 5) to 0.649 (item 2) on the single item level. With regard to intra-rater reliability, the ICC was 0.945 on the total score level, with Cohen’s weighted k ranging from 0.469 (item 5) to 0.883 (item1) on the single item level. Cronbach’s α value of all 5 items amounted to 0.746. Spearman’s rank correlation coefficient between OPTION^5^ and OPTION^12^ for Chinese versions was 0.660.

**Conclusions:**

The simplified Chinese version of the OPTION^5^ scale, developed using stringent translation procedures, demonstrated satisfactory psychometric characteristics. Specifically, inter- and intra-rater reliabilities were excellent, while criterion validity was moderate. The simplified Chinese version of the OPTION^5^ scale can be implemented in clinical settings to evaluate SDM of treatment during consultations between GPs and chronically ill patients.

**Supplementary Information:**

The online version contains supplementary material available at 10.1186/s12875-020-01335-2.

## Background

In recent decades, there has been a particular emphasis on the ways to increase patient involvement in treatment decisions. Placing patients at the center of care and enhancing patient autonomy have become essential milestones in the new approach to the improvement of the quality of medical care [1]. By definition, shared decision making (SDM) is a process where both the patient and health professional jointly make a decision based on the most comprehensive evidence about available treatment options and the patient’s values and preferences [2]. According to available evidence, apart from being an ethical principle (i.e., respecting patient’s autonomy and decision capacity), the SDM process increases participants’ knowledge, reduces decisional conflict, increases accuracy of risk perceptions [3], and promotes patients’ satisfaction with care [4]. Effective SDM has been shown to improve patients’ compliance and lead to better health results [5]. Furthermore, the results of several systematic reviews have confirmed that most patients prefer sharing decisions with physicians [6] and that physicians positively evaluate using SDM in their clinical practice [7].

However, upon an evaluation of clinicians’ communication ability to share information with patients and to promote patients’ involvement in decision making, there is a need to evaluate the degree of implementation of SMD in health care. In this respect, Observing Patient Involvement in Decision Making (OPTION), a frequently applied decision-making assessment scale during medical encounters [8], is an effective tool to objectively evaluate the interaction between clinicians and patients from the perspective of observers. The OPTION scale consists of 12 items (OPTION^12^) to be rated on a 5-point Likert scale (‘behavior not observed=0’; ‘behavior exhibited to a very high standard=4’) and evaluates the extent to which clinicians involve patients in medical decisions [9]. Using the data collected by using the OPTION scale, researchers can evaluate clinicians and patients’ decision-making process by directly observing their on-the-spot dialogues, or via analyzing relevant audio or video recordings [10]. To date, the OPTION^12^ scale has been translated into many languages including Chinese [11]. In 2013, Elywn et al. simplified OPTION^12^ into the OPTION^5^ scale where the items not belonging to the core components of SDM were deleted, so that only 5 items focused on the guidance and integration of patients’ preferences and assessing the extent of providers promoting patients’ involvement in decision making were retained [12]. The results of a comparative analysis of OPTION^5^ and OPTION^12^ demonstrated a good inter-rater agreement and significant positive correlation between the two versions of the scale [13]. The English version of OPTION^5^ (Additional file 1) showed a valid and reliable SDM estimate, as well as inter-rater reliability and intra-rater reliability on the item level [13].

At present, OPTION^5^ has been translated into several languages, including, among others, German and Dutch. For the German version of OPTION^5^, inter- and intra-rater reliability were both excellent on the total score level, which demonstrated a moderate concurrent validity using OPTON^12^ [14]. In the Dutch version, upon appropriate adjustments of the OPTION manuals, the inter-rater agreement of OPTION^12^ and OPTION^5^ ranged from good to excellent. There was also a high correlation between the total scores. Furthermore, OPTION^5^ with a wider score range systematically scored higher than OPTION^12^ [15].

As for the extent of SDM observed by OPTION scale, a systematic review assessed studies that used the OPTION^12^ scale to observe the extent to which health-care providers involve patients in decision making, and found that few providers consistently attempt to promote patient involvement, and even fewer adjust care to patient preferences [16]. In the English OPTION^5^, item scores in the PDA group were higher than in the usual care arm, which means observed a higher level of SDM in the PDA group than the usual care arm [13]. In the Dutch validation, OPTION^5^ seems to differentiate better between various levels of patient involvement [15].

With regard to China, while the assessment of SDM is made mostly from patients and clinicians’ points of view, the rating evaluated by observers is still in the initial stage. Huang verified the effect of the English OPTION^12^ in promoting SDM process between Chinese clinicians and patients with coronary heart disease [17]. However, other researches that would apply the Chinese version of OPTION^12^ have not been undertaken yet. In this context, following Prof. Elwyn’s consent, the present study aimed to establish a simplified Chinese version of Observer OPTION^5^ scale and to test its reliability and validity.

## Methods

### Translation process

The process of translating the Observer OPTION^5^ scale from English to Chinese was performed to reach cross-cultural equivalence [18]. The translation process consisted of the following nine steps: (1) *Preparation* (upon authorization and consent from the main developer of the OPTION^5^ scale); the plan of the translation process was established and sent to Prof. Elwyn for review; (2) *Forward translation*; two medical professionals, who were Chinese native speakers and were proficient in English, independently translated the English OPTION^5^ scale; (3) *Reconciliation*; upon an in-depth discussion, the team members including the two aforementioned translators compared the translated versions and merged them into one; (4) *Back translation*; two college English teachers familiar with medical research translated the Chinese version produced in Step (3) back into English; (5) *Back translation review;* the team members compared the back-translated versions with the original and highlighted discrepancies between them. The original, the Chinese reconciled translation, and two back-translated versions were sent to Prof. Elwyn for consultation and revision; (6) *Harmonization*; which was achieved after the team discussion based on Prof. Elwyn’s feedback; (7) *Rater training*; after the OPTION^5^ user manual was translated, evaluated, and revised into a final simplified Chinese version through team discussion, two raters were trained according to the translated OPTION^5^ user manual; (8) *Cognitive debriefing*; two raters independently rated consultation recordings from 5 patients in order to check understandability, interpretation, and cultural relevance of the translation; (9) *Proofreading*; the simplified Chinese OPTION^5^ scale was finally reviewed and calibrated through team discussion.

The final simplified Chinese OPTION^5^ scale and user manual are available from the corresponding author upon request. Table 1 shows the basic information about the key actors of the translation process.

**Table 1:** Basic information on the key actors involved in the translation process

Actor	Work undertaken	Educational degree	Professional	Title
A	1.Preparation; 2.Forward translation; 3.Reconciliation;4. Back translation review; 5. Harmonization;6. Rater training; 7.Proofreading	Ph.D.	General practice	Associate professor
B	1.Forward translation; 2. Reconciliation; 3.Back translation review; 4.Harmonization; 5.Rater training; 6.Proofreading	Ph.D.	General practice	Lecturer
C	1.Preparation; 2.Reconciliation; 3.Back translation review; 4.Harmonization; 5.Rater training; 6.Cognitive debriefing; 7.Proofreading	Postgraduate	General practice	–
D	1.Back translation; 2.Back translation review; 3. Harmonization; 4. Proofreading	Ph.D.	English Education	Lecturer
E	1. Back translation; 2.Back translation review; 3.Harmonization; 4. Proofreading	Ph.D.	English Education	Lecturer
F	1.Reconciliation; 2.Harmonization; 3.Rater training; 4.Cognitive debriefing; 5.Proofreading	Postgraduate	General practice	Clinician

### Setting and design

One rater (BX), who did not involve in the activities, observed with audio-recordings the consultations between GPs and chronically ill patients from May to June 2019 in a Beijing community health service center (CHSC). Meanwhile, demographic data of the patients and GPs were collected by the rater via information forms.

Inclusion criteria for the patients were as follows: (1) a chronic disease (mainly hypertension or other chronic diseases); (2) age above 18 years old; (3) Chinese-speaking; (4) facing a specific treatment decision; (5) willing to participate in the study; and (6) oral consent to be recorded. Patients with cognitive impairment or visiting their GP only for a medicine prescription were excluded. Inclusion criteria for GPs were as follows: (1) working at CHSC for over a year; (2) signing informed consent (including consent and audio-recording); (3) willing to participate in the study. A total of 10 GPs and 209 patients who met inclusion criteria were included in the final sample. A total of 209 audio-recordings were rated with the simplified Chinese version of OPTION^5^ scale.

### Sample size

With an estimated dropout of 20% consultations, an enough sample size was aimed to detect correlations above 0.5 with a power of 80% for the planned analyses. A sample size of *N* = 100 was finally determined.

In the present study, each rater had to achieve the target of observing no fewer than 10 consultations between each GP and patients with hypertension. A total of 10 GPs and 209 patients with chronic diseases (including hypertension, type-2 diabetes, hyperlipidemia, and other common chronic diseases) who met inclusion criteria were included.

### Rating process

A total of 209 audio-recordings were separately evaluated by both raters (ZY and BX) in order to assess inter-rater reliability of the simplified Chinese version of Observer OPTION^5^ from July to September 2019. One of the raters (BX) rated them a second time within one month from the first rating to assess intra-rater reliability.

### Data analysis

The data were analyzed using SPSS Statistics 22 (SPSS Inc., Chicago, IL). General demographic data of the patients and GPs were analyzed by descriptive statistics. All item scores and total scores of OPTION^5^ were statistically described. In general, intra-class correlation coefficient (ICC) can be used for quantitative or classified data, while the kappa coefficient method is suitable for classified data [19]. In the present study, inter- and intra-rater reliabilities were calculated using weighted Cohen’s kappa value on single item testing, and using ICC on the total score testing. Cronbach’s α was applied to evaluate the internal consistency of the scale. Strength of agreement of weighted Cohen’s Kappa statistics between 0.81–1.00 was classified as almost perfect, 0.61–0.80 as substantial, 0.41–0.60 as moderate, 0.21–0.40 as fair, 0.00–0.20 as slight, and < 0.00 as poor [20]. The results of ICC between 0.75–1.0 were classified as excellent, 0.6–0.74 as good, 0.40–0.59 as moderate, and 0–0.39 as poor [21]. Because there is a violation of normality, criterion validity was calculated using Spearman’s rank correlation to compare OPTION^5^ and OPTION^12^.

## Results

### Basic characteristics of the GPs and patients

All 10 observed GPs were female, aged 28–64 years old (mean age = 42.40 ± 9.69), with over 10 years of work experience (see Table 2).

**Table 2:** Characteristics of the GPs (*N* = 10)

Category	Subcategory	N	Category	Subcategory	N
Age (years)	<30	1	Professional positions	Junior-level title	1
	30~	3		Middle-level title	2
	40~	6		Senior-level title	7
Education	Bachelor	5	Work experience (years)	<10	2
	Master	4		10~	3
	Doctor	1		20~	5

A total of 1969 patients were involved during the observed period, and 209 of the 1969 patients participated in the decision making regarding a chronic disease treatment. The average age of the 209 patients was in the range of 41–87 years old (mean 64.42 ± 8.64), and the average consultation time ranged from 1.0 to 17.8 min (mean 5.13 ± 3.25). Of all consultations, 99 focused on the decisions regarding hypertension, 39 concerning Type 2 diabetes, 34 concerning hyperlipidemia, 20 concerning another chronic disease, and 17 concerning multiple other chronic diseases (see Table 3).

**Table 3:** Characteristics of the patients (*N* = 209)

Classification		N	%
Gender	Male	92	44.0
	Female	117	56.0
Age (years)	41 ~ 49	13	6.2
	50 ~ 59	41	19.6
	60 ~ 69	103	49.3
	70 ~ 87	52	24.9
Education^a^	Junior high school and below	35	21.2
	Senior high school/technical secondary school	39	23.6
	Junior college and above	91	55.2
Diseases involved in decision making	Hypertension	99	47.4
	Type 2 diabetes	39	18.7
	Hyperlipidemia	34	16.3
	One other chronic disease	20	9.6
	Other multiple chronic diseases	17	8.1
Consulting duration (min)	0.1 ~ 3.7	89	42.6
	3.8 ~ 7.5	86	41.1
	7.6 ~ 11.3	21	10.0
	11.4 ~ 15.0	7	3.3
	≥15.1	6	2.9

^a^165 effective respondents

### The results of rating item of simplified Chinese version of OPTION^5^

The rating results from Rater 1 and Rater 2 showed that the scores of the items were mainly concentrated on 1 and 2; the frequencies of the scores are reported in Tables 4 and 5.

**Table 4:** Item frequencies and median score of Rater 1

Item	No effort (%)(0)	Minimal effort (%)(1)	Moderate effort (%)(2)	Skilled effort (%)(3)	Exemplary effort (%)(4)	Median (Range)
Item 1	0 (0)	92 (44.0)	101 (48.3)	16 (7.7)	0 (0)	2 (1 ~ 2)
Item 2	81 (38.8)	82 (39.2)	40 (19.1)	6 (2.9)	0 (0)	1 (0 ~ 1)
Item 3	4 (1.9)	25 (12.0)	102 (48.8)	78 (37.3)	0 (0)	2 (2 ~ 3)
Item 4	14 (6.7)	120 (57.4)	73 (34.9)	2 (1.0)	0 (0)	1 (1 ~ 3)
Item 5	12 (5.7)	130 (62.2)	65 (31.1)	2 (1.0)	0 (0)	1 (1 ~ 3)
Total score						35 (25 ~ 45)^a^

^a^:rescaled to a total score of 0 to 100

**Table 5:** Item frequencies and median score of Rater 2

Item	No effort (%)(0)	Minimal effort (%)(1)	Moderate effort (%)(2)	Skilled effort (%)(3)	Exemplary effort (%)(4)	Median (Range)
Item 1	0 (0)^a^/ 0 (0)^b^	78 (37.3)^a^/ 85 (40.7)^b^	111 (53.1)^a^/ 103 (49.3)^b^	20 (9.6)^a^/ 21 (10.0)^b^	0 (0)^a^/ 0 (0)^b^	2 (1 ~ 2)^a^/ 2 (1 ~ 2)^b^
Item 2	71 (34.0)^a^/ 77 (36.8)^b^	116 (55.5)^a^/ 103 (49.3)^b^	21 (10.0)^a^/ 26 (12.4)^b^	1 (0.5)^a^/ 3 (1.4)^b^	0 (0)^a^/ 0 (0)^b^	1 (0 ~ 1)^a^/ 1 (0 ~ 1)^b^
Item 3	3 (1.4)^a^/ 2 (1.0)^b^	68 (32.5)^a^/ 57 (27.3)^b^	92 (44.0)^a^/ 94 (45.0)^b^	46 (22.0)^a^/ 56 (26.8)^b^	0 (0)^a^/ 0 (0)^b^	2 (1 ~ 2)^a^/ 2 (1 ~ 3)^b^
Item 4	16 (7.7)^a^/ 9 (4.3)^b^	149 (71.3)^a^/ 144 (68.9)^b^	44 (21.1)^a^/ 55 (26.3)^b^	0 (0)^a^/ 1 (0.5)^b^	0 (0)^a^/ 0 (0)^b^	1 (1 ~ 1)^a^/ 1 (1 ~ 2)^b^
Item 5	39 (18.7)^a^/ 12 (5.7)^b^	142 (67.9)^a^/ 142 (67.9)^b^	27 (12.9)^a^/ 54 (25.8)^b^	1 (0.5)^a^/ 1 (0.5)^b^	0 (0)^a^/ 0 (0)^b^	1 (1 ~ 1)^a^/ 1 (1 ~ 2)^b^
Total score*						30 (25 ~ 40)^a^/ 30 (25 ~ 45)^b^

^a^ first rating; ^b^ second rating, one month after the first rating; *rescaled to a total score of 0 to 100

### Reliability of the simplified Chinese version of OPTION^5^

#### Inter-rater reliability

On the total score level, inter-rater ICC of the simplified Chinese version of OPTION^5^ was 0.859; 95% confidence interval was 0.724–0.917 (*p* < 0.001). Weighted Cohen’s kappa value of OPTION^5^ ranged from 0.376 (item 5) to 0.649 (item 2) on the single item level (see Table 6).

**Table 6:** Inter-rater weighted Cohen’s kappa value by single item

Item	Kappa(N = 209)	Agreement	Z value	95% CI	*p*
Item 1	0.508	Moderate	9.179	0.411 ~ 0.606	<0.001
Item 2	0.649	Substantial	13.212	0.566 ~ 0.731	<0.001
Item 3	0.531	Moderate	11.826	0.448 ~ 0.614	<0.001
Item 4	0.531	Moderate	10.335	0.423 ~ 0.639	<0.0 01
Item 5	0.376	Fair	8.853	0.275 ~ 0.477	<0.001

#### Intra-rater reliability

On the total score level, the intra-rater ICC of the simplified Chinese version of OPTION^5^ was 0.945; 95% confidence interval was 0.904–0.965 (*p* < 0.001). The weighted Cohen’s kappa value of OPTION^5^ ranged from 0.469 (item 5) to 0.883 (item1) on the single item level (see Table 7).

**Table 7:** Intra-rater weighted Cohen’s kappa value by single item

Item	Kappa(N = 209)	Agreement	Z value	95% CI	*p*
Item 1	0.883	Almost perfect	16.083	0.827 ~ 0.938	<0.001
Item 2	0.827	Almost perfect	15.939	0.754 ~ 0.900	<0.001
Item 3	0.843	Almost perfect	16.675	0.786 ~ 0.901	<0.001
Item 4	0.743	Substantial	13.701	0.653 ~ 0.833	<0.001
Item 5	0.469	Moderate	10.671	0.360 ~ 0.578	<0.001

#### Internal consistency

Cronbach’s α value of 5 items of the simplified Chinese version of OPTION^5^ was 0.746, indicating that there was a good internal consistency in these 5 items in the scale.

### The validity of the Chinese version of OPTION^5^

**Fig. 1:**
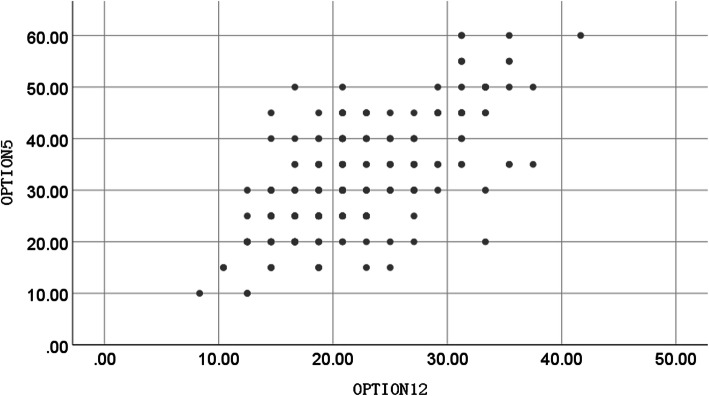
The scatterplot of the total scores of OPTION^12^ and OPTION^5^

Spearman’s rank correlation coefficient between OPTION^5^ and OPTION^12^ for the Chinese versions was 0.660 (*p* < 0.001), indicating that there was a moderate positive correlation between the two scales (Fig. 1).

## Discussion

In the present study, the original OPTION^5^ was translated into Chinese and cross-culturally adapted for further testing of its reliability and validity. Two raters independently assessed the simplified Chinese version of OPTION^5^ using the audio recording acquired from the consultations between GPs and chronically ill patients at CHSC.

### The validation of the simplified Chinese version of OPTION^5^

The results showed that the simplified Chinese version of OPTION^5^ has satisfactory psychometric properties. The scale was found to be reliable on the total score level and to have a good internal consistency among 5 items of the Chinese version. On the single item level, inter-rater reliability coefficient ranged from fair to substantial (kappa = 0.376–0.649), and intra-rater reliability coefficient ranged from medium to excellent (kappa = 0.469–0.883). Furthermore, a moderate positive correlation between Observer OPTION^5^ and OPTION^12^ also showed a good concurrent validity of the simplified Chinese OPTION^5^. These results provide convincing evidence about the validity and reliability of the scale and are consistent with the results of previous studies [13, 14]. Therefore, the simplified Chinese version of OPTION^5^ produced in the present study can be effectively used to measure GPs facilitating patients’ participation in the SDM process and to evaluate the decision-making process in clinical practice and health service research from an observer’s perspective.

On the single item level, item 5 was the only item that had the lowest levels of inter-rater agreement (kappa = 0.376). This suggests a poor consistency of two raters on item 5. Consultations of longer duration may lead to reduced inter-rater agreement [13]. In further research, improving item specificity, training raters extensively, and providing more guidance may help to improve inter-rater consistency [13].

On the total score level, compared to inter-rater reliability in the first testing of the English version (ICC = 0.67) [13] and the psychometric testing in the DUTCH version [15] (K = 0.68), our result on inter-rater reliability was relatively higher (ICC = 0.859). This outcome may be related to the single clinical decision making involved in the consultation. In the present study, most doctor-patient interactions focused on only one problem in treatment. In previous studies, however, there may have been some ambiguity during clinical consultations, or more decisions needed to be made by clinicians and patients. This may have led to a relatively low consistency of studies that used the English version of the scale. In addition, we found an excellent intra-rater reliability on the total score level, which is consistent with the results of testing the German version of the scale [14].

### The observed low SDM extent and its potential causes

In addition to helping objectively assess SDM process, the OPTION^5^ scale also provides a general estimate of the involvement of two parties in a consultation [14].

From the perspective of single item, the scores of five studied items on inter-rater and intra-rater reliabilities were mostly concentrated on 1–2 points, which resulted in the left deviation of the distribution of the scores, indicating that GPs promotes and facilitates patients’ involvement to a low extent. This outcome has verified the conclusions in English and German studies since it is in line with the research results [13, 14]. Several possible reasons may explain the observed low level of SDM. First, none of the observed GPs were involved in SDM training. According to the systematic review of previous studies by using OPTION^12^, untrained healthcare providers also scored lower points [16]. The continuous training and education of GPs’ positive attitudes towards SDM, enhancement of their SDM theoretical background, and cultivation of ability to transfer SDM knowledge into routine clinical practice are the major measures to facilitate SDM process in consultation. Second, insufficient consultation time may be another cause. As can be analyzed from our other studies, during consultations for chronic diseases, the GPs usually spend more time in discussing with the patients seeking for specific treatment decision than with those merely for medicine prescription (median time = 4.4: 2.0 min, *P* < 0.05) [22]. However, due to the heavy workload in Beijing community, which hinders substantial communication and interaction between GPs and patients, GPs’ consultation time is dramatically not enough. Training more GPs or re-employing competent retired GPs in primary care is a solution to alleviate workload and increase consultation time [23]. Third, patients’ willingness to participate in decision making also needs to be guided and educated.

In addition, the rare observation of behavior captured by a specific item is likely to obtain a lower kappa score [24]. The item 2 on inter-rater and intra-rater reliabilities, namely, “The clinician reassures the patient or re-affirms that the clinician will support the patient to become informed or deliberate about the options. If the patient states that they have sought or obtained information prior to the encounter, the clinician supports such a deliberation process”, was the least observed in our study. The same item has been reported as problematic in the English OPTION^5^ scale [13]. The raters assessed the interactions between GPs and patients by means of recordings, which imply that GPs’ body languages in supporting patients could be neither observed nor positively scored.

#### Strengths and limitations

Overall, the results of the present study demonstrated that the simplified Chinese version of OPTION^5^ is a feasible, reliable, and effective observer rating scale. The scale can be effectively used to evaluate SDM in doctor-patient communication, clinicians’ communication skills, as well as the level of clinicians’ promotion of patients’ involvement in decision making. To date, it is the shortest available observer rating scale for SDM [13].

However, the present study has several limitations. First, due to all participating GPs were female in our study, we could not sure whether there was a gender bias in the findings. According to available evidence, compared to female clinicians, their male counterparts are more likely to be doctor-centered and are less likely to guide patients to participate in decision making about treatment [25–27]. Therefore, in future research, it would be advisable to include a more gender-balanced sample of GPs.

Second, the simplified Chinese version of OPTION^5^ was conducted at a single medical center (CHSC), for mostly three chronic diseases. The scope of application of the scale was somewhat limited. Therefore, in future studies, it would be necessary to apply the scale in more diverse medical settings and for other chronic diseases [28].

Third, the rating materials were audio recording, and one of the two raters observed the nonverbal aspects of the interaction between GPs and patients, which may have led to discrepancies between the two raters.

Fourth, although the researcher did not participate in the interactions when observing the consultations, it was possible that the presence of the researcher influenced the behaviors of GPs and patients.

Finally, while the simplified Chinese OPTION^5^ is generally effective and reliable, in future research, improving single item specificity and providing more guidance may be needed.

## Conclusions

In the present study, the simplified Chinese version of OPTION^5^ was developed following stringent translation procedures. The results of testing the simplified Chinese OPTION^5^ scale showed that the scale has satisfactory psychometric characteristics. Overall, it can be concluded that the scale can be meaningfully used to evaluate the decision-making process regarding the treatment of chronic diseases in community clinical practice. Furthermore, this scale will contribute to providing effective information on clinical practices in China. However, further testing of the scale is recommended, particularly before applying it on other occasions or other patient groups that those targeted in the present study.

## Supplementary Information

**Additional file 1.** The English version of the Observer OPTION^5^ scale.

## Data Availability

The datasets used and/or analysed during the current study available from the corresponding author on reasonable request.

## Abbreviations

SDM: Shared decision making
OPTION: Observing Patient Involvement in Decision-Making
ICC: intra-class correlation coefficient
GP: General practitioner
CHSC: Community health service center
