# Supplementary material for: Psychometric properties of the simplified Chinese version of the observer OPTION5 scale
Source: BMC Fam Pract. 2020 Dec 6;21:263. doi: 10.1186/s12875-020-01335-2 (PMC7720385; doi:10.1186/s12875-020-01335-2)
Supplement: Supplementary file 1 — Additional file 1. The English version of the Observer OPTION5 scale. [file 12875_2020_1335_MOESM1_ESM.pdf]

The Observer OPTION [5] Measure -Score Sheet

Date **Number / Name**

**Item 1:** For the health issue being discussed, the clinician **draws attention to or confirms** that alternate treatment or management options exist or that the need for a decision exists. If the patient rather than the clinician draws attention to the availability of options, the clinician responds by agreeing that the options need deliberation.<br>0 = No effort 1 = Minimal effort 2 = Moderate effort 3 = Skilled effort 4 = Exemplary effort
**Item 2:** The clinician reassures the patient or re-affirms that the clinician **will support the patient to become informed or deliberate** about the options. If the patient states that they have sought or obtained information prior to the encounter, the clinician supports such a deliberation process.<br>0 = No effort 1 = Minimal effort 2 = Moderate effort 3 = Skilled effort 4 = Exemplary effort
**Item 3:** The clinician **gives information or checks understanding about the options** that are considered reasonable (this can include taking no action), to support the patient in comparing alternatives. If the patient requests clarification, the clinician supports the process.<br>0 = No effort 1 = Minimal effort 2 = Moderate effort 3 = Skilled effort 4 = Exemplary effort
**Item 4:** The clinician makes an effort to **elicit the patient's preferences** in response to the options that have been described. If the patient declares their preference(s), the clinician is supportive.<br>0 = No effort 1 = Minimal effort 2 = Moderate effort 3 = Skilled effort 4 = Exemplary effort
**Item 5:** The clinician makes an **effort to integrate the patient's elicited preferences** as decisions are made. If the patient indicates how best to integrate their preferences as decisions are made, the clinician makes an effort to do so.<br>0 = No effort 1 = Minimal effort 2 = Moderate effort 3 = Skilled effort 4 = Exemplary effort

Scoring Summary (see Manual for details).

Score	Description
0 = No effort	Zero effort observed.
1 = Minimal effort	Effort to communicate could be implied or interpreted.
2 =Moderate effort	Basic phrases or sentences used.
3 = Skilled effort	Substantive phrases or sentences used.
4 = Exemplary effort	Clear, accurate communication methods used.
